# Microencapsulation of Probiotics by Oil-in-Water Emulsification Technique Improves Cell Viability under Different Storage Conditions

**DOI:** 10.3390/foods12020252

**Published:** 2023-01-05

**Authors:** Sebastião Ânderson Dantas da Silva, Leonam da Silva Pereira Batista, Dara Souza Diniz, Sara Sayonara da Cruz Nascimento, Neyna Santos Morais, Cristiane Fernandes de Assis, Thaís Souza Passos, Francisco Canindé de Sousa Júnior

**Affiliations:** 1 Postgraduate Program in Nutrition, Health Sciences Center, Federal University of Rio Grande do Norte, Natal 59078-970, RN, Brazil; 2 Department of Nutrition, Health Sciences Center, Federal University of Rio Grande do Norte, Natal 59078-970, RN, Brazil; 3 Department of Pharmacy, Health Sciences Center, Federal University of Rio Grande do Norte, Natal 59012-570, RN, Brazil; 4 Postgraduate Program in Biotechnology-RENORBIO, Federal University of Rio Grande do Norte, Natal 59078-970, RN, Brazil

**Keywords:** *Lactobacillus acidophilus*, *Lactiplantibacillus plantarum*, encapsulation, porcine gelatin, storage stability

## Abstract

Probiotics are associated with health benefits to the host. However, their application can be limited due to a decrease in cell viability during processing, storage, and passage through the gastrointestinal tract. Microencapsulation is a simple and efficient alternative to improve the physical protection and stability of probiotics. The present study aimed to produce and characterize alginate or gelatin-based microparticles containing *Lactobacillus acidophilus* NRRL B-4495 or *Lactiplantibacillus plantarum* NRRL B-4496 by oil-in-water (O/W) emulsification and to evaluate the stability under storage conditions. The results showed that *L. acidophilus* and *L. plantarum* encapsulated in gelatin (LAEG and LPEG) presented diameters of 26.08 ± 1.74 μm and 21.56 ± 4.17 μm and encapsulation efficiencies of 89.6 ± 4.2% and 81.1 ± 9.7%, respectively. However, those encapsulated in alginate (LAEA and LPEA) showed an encapsulation efficiency of <1.0%. Furthermore, LAEG was stable for 120 days of storage at 5 °C and 25 °C. Therefore, encapsulation in gelatin by O/W emulsification is a promising strategy for protecting and stabilizing probiotic bacteria, enabling future application in foods.

## 1. Introduction

Probiotics are live microorganisms that confer a health benefit to the host when administered in adequate amounts [1]. Probiotics can prevent inflammatory diseases and allergic disorders, decrease the incidence of diarrhea, control infections, act as antibiotics, protect against colon and bladder cancer, and treat necrotizing enterocolitis, acute respiratory tract infections, and infantile colic [2].

It is widely accepted that the number of viable probiotic cells present in the food matrix must reach the minimum concentration of 10^6^–10^7^ colony forming units (CFU) per gram (g) or milliliter (mL) to promote health benefits to the host [3]. However, many strains may show considerable loss of viability during storage or under adverse conditions in the gastrointestinal tract (GIT) such as acidic stomach pH, digestive enzymes, and bile salts [4,5].

Microencapsulation is a simple and efficient solution to improve the survival of these microorganisms in food products during processing, storage, and passage through the GIT [6]. It is a strategy capable of promoting the protection of bioactive substances against extrinsic factors (temperature, pH, presence of oxygen and light, enzymatic digestion) and preventing probiotic multiplication in food and changes in its sensory attributes [7]. Furthermore, encapsulation can provide site-specific release at a controlled rate and preserve or enhance the effect of encapsulated substances [8].

Many encapsulating agents such as pectin, alginate, carrageenan, chitosan, whey proteins, gelatin, and starch have been used to encapsulate probiotic bacteria and other bioactive components [3,8,9]. Alginate is a biopolymer derived from brown algae (Kelp), mainly formed by β-D-mannuronic and α-L-guluronic acids [10]. This polymer has a simple structure, lacks toxicity, biocompatibility, low cost, and is a widely used matrix to encapsulate probiotics. Gelatin is a water-soluble polymer produced by the partial hydrolysis of collagen [11]. It is formed by a complex mixture of polypeptide chains with different chains, which can also be used in probiotic encapsulation [10]. It is helpful as a thermally reversible gelling agent for encapsulation due to its amphoteric nature [12].

Probiotics can be encapsulated using various methods such as spray drying, coacervation, ionic gelation, and emulsification [13,14,15]. Water-in-oil (W/O) or water-in-oil-in-water (W/O/W) emulsification has been used in several studies to protect probiotics using different materials [2]. The oil-in-water (O/W) emulsification technique is recommended to promote probiotic protection during food manufacturing and storage [16]; however, few studies have focused on this technique [17,18,19]. In addition, converting a liquid emulsion into powder using drying techniques can facilitate its applications in foods [20].

Therefore, this study aimed to produce and characterize microparticles based on alginate or gelatin loaded with *Lactobacillus acidophilus* NRRL B-4495 and *Lactiplantibacillus plantarum* NRRL B-4496 obtained by O/W emulsification and to evaluate their stability in storage conditions.

## 2. Materials and Methods

### 2.1. Materials

De Man, Rogosa, and Sharpe (MRS) broth was purchased from Difco^®^ (Detroit, MI, USA). Porcine gelatin (Type A), sodium alginate, and Tween 20 were obtained from Sigma-Aldrich^®^ (St. Louis, MO, USA). Glycerol was supplied by Synth, peptone by Vetec^®^ (St. Louis, MO, USA), and bacteriological agar by Kasvi^®^ (Campina, Brazil). Corn oil (Liza^®^) was obtained from a supermarket in Natal, RN, Brazil.

### 2.2. Strains

*Lactobacillus acidophilus* NRRL B-4495 (LA) and *Lactiplantibacillus plantarum* NRRL B-4496 (LP) probiotic strains were provided by the culture collection of the U.S. Department of Agriculture (ARS Culture Collection-NRRL, Peoria, Illinois). Strains were stored at −20 °C in microtubes containing MRS broth and 15% glycerol (*w/v*).

### 2.3. Activation of Microorganisms

First, 2 mL aliquots of the glycerol stock culture were transferred to 100 mL of MRS broth containing 10 mL of 200 mM phosphate buffer, pH 6.5. This initial cultivation was carried out at 37 °C for 24 h. Then, 2 mL of the initial culture was transferred to 100 mL of MRS broth and 10 mL of phosphate buffer and incubated at 37 °C for 17 hours. Next, the cells were centrifuged at 4000 rpm for 15 min (SL 701-Solab^®^, Piracicaba, Brazil), washed with 0.9% NaCl solution (*w/v*), and subjected to a second centrifugation to obtain the pellet used in the encapsulation procedures.

### 2.4. Encapsulation of Probiotics

Probiotics were encapsulated using an oil-in-water (O/W) emulsification technique based on Quintana et al. [17] with modifications. The encapsulates were formulated using sodium alginate or porcine gelatin as encapsulating agents.

The oil phase (OP) consisted of 4 g of corn oil. Two aqueous phases were formulated: aqueous phase 1 (AP1–100 mL) containing 0.5% (*w/v*) Tween 20 and 4% (*w/v*) encapsulating agent solubilized in distilled water. Aqueous phase 2 (AP2–50 mL) containing 0.5% (*w/v*) Tween 20 solubilized in autoclaved distilled water at pH 5.5.

The probiotic pellet was resuspended in AP1, and the pH of the mixture was adjusted to a value of 5.5 with 1% HCl or 1% NaOH. After resuspension of the probiotic, 1 mL of each AP1 was collected for initial probiotic enumeration (N_0_—Non-encapsulated probiotics).

The AP1 was homogenized with the oil phase under ultra-dispersion (5000 rpm) (Ultra-Turrax, T18 basic, IKA^®^, Staufen, Germany) for 7 min to promote emulsion formation. Then, AP2 was dispersed in the emulsion obtained using the same conditions above-mentioned. Four encapsulated samples were subsequently obtained, and then dried by lyophilization (LioTop L101, LIOBRAS, Vila Alpes, Brazil) at −57 °C and a pressure of 43 μHg. After lyophilization, 1.0 g of the powders were used to perform serial dilutions and count the probiotic (N), which was later used to determine the encapsulation efficiency.

### 2.5. Characterization of the Obtained Formulations

#### 2.5.1. Scanning Electron Microscopy (SEM)

The powder particles were placed on carbon tape fixed on stubs and metalized in gold and palladium for 75 s. Analyses were performed using a MEV-VEGA3-SEM microscope (Tescan Analytics^®^, Brno, Czech Republic) at different magnifications, high vacuum, and 5–20 kV voltage.

#### 2.5.2. Laser Diffraction

The mean diameter and polydispersity index were determined using 5 mg of LAEG and LPEG formulations dispersed separately in 4 mL of acetone (PA) and 10 mg of LAEA and LPEA formulations dispersed in 4 mL of dimethylsulfoxide. The dispersions were analyzed in NanoBrook ZetaPlus Zeta Potential Analyzer equipment^®^ (Brookhaven Instruments, Holtsville, NY, USA) using the Brookhaven Instruments-ZetaPALS Particle Sizing Software program^®^ (Holtsville, NY, USA) [21].

#### 2.5.3. Zeta Potential

The zeta potential was determined using a STABINO II particle charge titration device (Colloid Metrix) according to the methodology described by Morais et al. [21]. First, the sample was diluted in ultrapure water. The zeta potential measurement with pH variation (similar to a titration) consisted of individually adding aliquots (10 μL) of a strong acid (0.1 M HCl) or a strong base (0.025 M NaOH).

#### 2.5.4. Fourier Transform Infrared Spectroscopy (FTIR)

The formulations and raw constituents (alginate, gelatin, and Tween 20) were homogenized with potassium bromide (KBr), macerated, and pressed to obtain tablets. They were recorded in transmittance and with a mid-infrared region of 400 to 4000 cm^−1^ using a Shimadzu^®^ (Kyoto, Japan) FTIR-8400S IRAFFINITY-1 series spectrometer and the IRSOLUTION^®^ (Kyoto, Japan) version 1.60 software program, with 32 scans and resolution of 4 cm^−1^ [22].

#### 2.5.5. X-ray Diffraction

The encapsulating agents (sodium alginate and gelatin) and formulations obtained were analyzed in a Bruker^®^ (Billerica, MA, USA) high-resolution X-ray diffractometer (D2Phaser) equipped with a Lynxeye detector with copper radiation (CuKα, λ = 1.54 Å), Ni filter, current of 10 mA, and voltage of 30 kV. The materials were analyzed in the range of 2θ 10–100, with a divergent slit of 0.6 mm, a central slit of 1 mm, a convergent slit of 0.02°, and an acquisition time of 0.1 s [22].

### 2.6. Enumeration of Viable Cells and Determination of Encapsulation Efficiency

The final AP1 suspension was collected, followed by successive dilutions in peptone saline water to evaluate the initial viability of the probiotic (N_0_). Then, 0.1 mL of the dilutions were inoculated on MRS agar and incubated at 37 °C for 72 hours according to the methodology described by Ribeiro et al. [5].

The release of probiotics from the particles was performed according to the protocol described by Sheu et al. [23] with modifications. For this, 1 g of each encapsulated material was added to 9 mL of 0.1 M potassium phosphate buffer (pH 7.5), stirred at 150 rpm for 1 minute, and left at 37 °C for 10 minutes. Then, the mixture was stirred at 150 rpm for 1 minute and subjected to successive dilutions in peptone saline water. The dilutions were then inoculated on MRS agar and incubated as described for N_0_. The encapsulation efficiency (EE) was calculated according to Equation (1):

EE (%) = (N/N_0_) × 100 (1)

where EE is the encapsulation efficiency (%); N is the number of viable cells in the encapsulates; and N_0_ is the number of viable cells in AP1 (non-encapsulated probiotics).

### 2.7. Dispersibility

The water dispersibility of gelatin-based encapsulates was evaluated according to the methodology previously described by Paula et al. [4]. The dispersibility results were determined according to Equation (2):

Dispersibility (%) = [(m_1_ × 2)/m_0_] × 100 (2)

where m_1_ is the mass (g) in the 25 mL aliquot of the supernatant after drying (g) and m_0_ is the initial sample (g) mass incorporated in 50 mL of distilled water.

### 2.8. Thermogravimetry (TG) and Differential Thermal Analysis (DTA)

TG/DTA tests were performed to investigate the thermal degradation behavior of raw materials (corn oil, Tween 20, and gelatin), lyophilized free probiotics, and gelatin-based encapsulated probiotics (LAEG and LPEG). Samples were evaluated in a Shimadzu^®^ DTG-60 thermal analyzer, with a heating rate of 10 °C per minute, in a temperature range from 29 °C to 800 °C in a nitrogen atmosphere with a flow rate of 50 mL/min [21].

### 2.9. Evaluation of the Stability of Encapsulated Probiotics during Storage

The stability of gelatin-based encapsulates containing *L. acidophilus* (LAEG) was evaluated for 120 days of storage based on Matos-Jr et al. [14], with modifications. After lyophilization, the formulation was weighed (3 g) and randomly placed in sterile, transparent plastic containers with lids and kept at room temperature (25 ± 2 °C) in a Tecnal^®^ (Piracicaba, Brazil) TE-392/170L bacteriological oven, and under refrigeration (5 ± 2 °C) in an Electrolux^®^ (Stockholm, Sverige) refrigerator (Cycle Defrost Duplex DC50).

Viable cells were counted and X-ray diffraction was performed immediately after lyophilization (day 0) and after 7, 15, 30, 45, 60, 90, and 120 days to investigate cell viability and the structural characteristics of the particles. Viability was investigated following the procedure described in Section 2.6 and X-ray diffraction according to Section 2.5.5.

### 2.10. Statistical Analysis

All experiments were performed as three replicates (*n* = 3). The obtained results were expressed as the mean (standard deviation). Analysis of variance (ANOVA) was performed with Tukey’s post-test considering a *p*-value < 0.05 as statistically significant and using the Statistica 8.0 software program (StatSoft Inc., Tulsa, OK, USA).

## 3. Results and Discussion

### 3.1. Microparticle Characterization

#### 3.1.1. Scanning Electron Microscope

**Figure 1:**
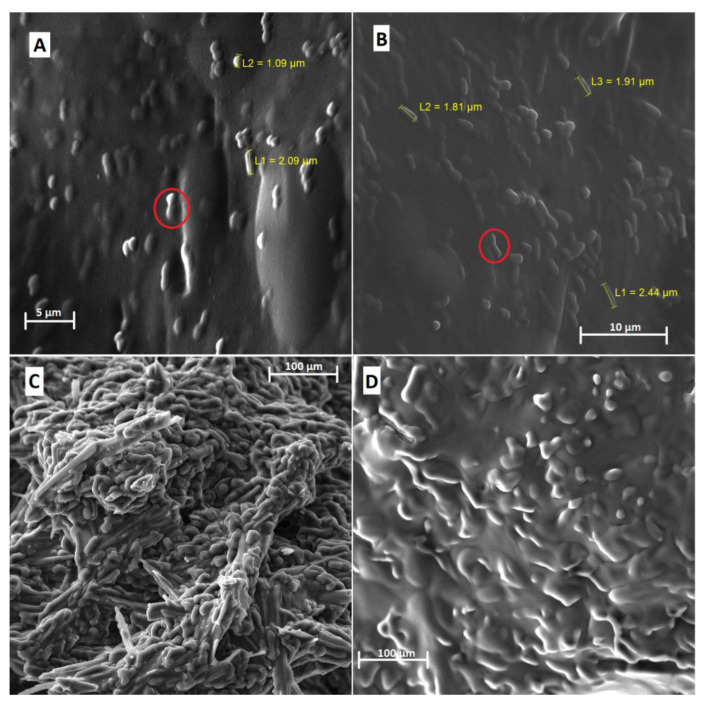
Scanning electron microscopy of the formulations obtained by O/W emulsification. (**A**) LAEG—*L. acidophilus* in gelatin with a magnitude of 7.01 kx; (**B**) LPEG—*L. plantarum* in gelatin with a magnitude of 6.35 kx; (**C**) LAEA—*L. acidophilus* in alginate with a magnitude of 542×; (**D**) LPEA—*L. plantarum* in alginate with a magnitude of 565×. Red circles indicate the presence of microbial cells.

The micrographs of the gelatin-based formulations containing probiotics showed irregular shapes, which can be attributed to lyophilization (Figure 1A,B). In addition, the presence of rod-shaped bacterial cells, which are homogeneously distributed and trapped in the structure, indicates the protection of probiotics.

The results obtained in the present study are similar to those described by Singh et al. [15] in encapsulating *L. rhamnosus* GG LMG 18,243 by the water-in-water emulsification (colloidal dispersions of an aqueous solution into another aqueous phase, which are thermodynamically incompatible) using carboxymethylcellulose and gelatin. After lyophilization, these authors also observed foam-like microstructures with porous appearance, alveolar morphology, and bacteria on the surface of the pores [15].

For the alginate-based formulations (Figure 1C,D), it is possible to observe the particles with morphological aspects characterized by irregular shapes, smooth surfaces, and shrinkage of the structures. However, the presence of microorganisms under the surface was not observed (as observed in gelatin-based particles).

The porous aspect of alginate-based microparticles is well-demonstrated in the literature [24]. Furthermore, sublimation drying of ice crystals under vacuum also forms dry porous particles [25]. However, based on the micrographs obtained in the present study, it was impossible to observe the presence of porosity in the structures, possibly due to increased moisture in the alginate-based microparticles, which showed high hygroscopicity.

#### 3.1.2. Laser Diffraction

The mean particle diameters for the gelatin-encapsulated groups (LAEG and LPEG) were 26.08 ± 1.74 µm and 21.56 ± 4.17 µm, with polydispersity indices of 0.5 ± 0.0 and 0.6 ± 0.1, respectively (Table 1). The low polydispersity value (<1) indicates the homogeneity of the particles obtained [26].

Arslan et al. [27] obtained a particle size of 21.38 µm for the gelatin-based formulations by microencapsulating *Saccharomyces cerevisiae* var. *boulardii* using different wall materials followed by spray-drying. However, it is important to highlight that the technique and surfactant used are essential for obtaining particles with a smaller diameter.

Sodium alginate encapsulated particle sizes were 5.24 ± 1.32 µm for LAEA and 5.52 ± 0.45 µm for LPEA, showing smaller values than the gelatin-based particles. In addition, LAEA and LPEA showed lower polydispersity indices (Table 1). Martin et al. [28] encapsulated *L. fermentum* CECT5716 in alginate or alginate and unmodified starch by W/O emulsification and internal gelation, obtaining sizes ranging from 30 to 60 μm. This difference may be related to the emulsification type and the surfactant used in the encapsulation process.

The application of encapsulated probiotics in the food industry is a challenge, mainly due to the microbial cells that lead to large particles, negatively affecting the sensory attributes of foods [10]. On the other hand, Martin et al. [28] reported that the acceptable particle size for food application should not exceed 80 μm to avoid a negative sensory effect. Therefore, the O/W emulsification technique and the surfactant used in the present study provide smaller particles and a polydispersity index. These results can enable applying encapsulated probiotics in food products without affecting their sensory attributes.

#### 3.1.3. Zeta Potential

The zeta potential results may be used to predict the stability of encapsulated materials in colloid systems. Colloid systems with potential values +10 to +20 or −10 to −20 mV are relatively stable, and values > +30 or < −30 mV are highly stable [29].

It is possible to observe that the alginate-based microencapsulates presented a negative charge in the studied range and presented stability in the pH range between 7.0 and 10.0 (Figure S1—Supplementary Materials). On the other hand, the porcine gelatin-based encapsulates presented positive surface charges at acidic pH. These formulations showed relatively stable (+13 mV) or moderately stable (+21 mV) charges over a wide pH range, ranging from 2.7 to 6.0. Particles with positive zeta potential are desirable as they facilitate cell membrane interaction, contributing to the controlled release of the compounds of interest [30].

#### 3.1.4. Fourier Transform Infrared Spectroscopy (FTIR)

**Figure 2:**
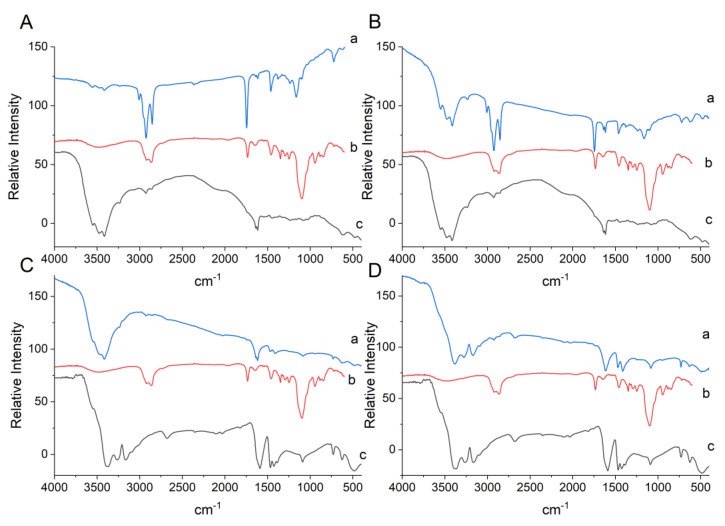
FTIR spectra of the encapsulates obtained by O/W emulsification. (**A**) LAEG—*L. acidophilus* in gelatin (**a**); Tween 20 (**b**); porcine gelatin (**c**). (**B**) LPEG—*L. plantarum* in gelatin (**a**); Tween 20 (**b**); porcine gelatin (**c**). (**C**) LAEA—*L. acidophilus* in alginate (**a**); Tween 20 (**b**); alginate (**c**). (**D**) LPEA—*L. plantarum* in alginate (**a**); Tween 20 (**b**); sodium alginate (**c**).

The Tween 20 spectrum exhibited a spectral band in the region of 3511 cm^−1^ for binding (O–H). The bands at 2920 cm^−1^ and 2866 cm^−1^ correspond to the vibration stretching of asymmetric and symmetric (C–H) alkyl bonds. Stretching of the ester group occurred at 1735 cm^−1^ (C=O), 1460 cm^−1^ for methylene (CH_2_), and at 1248 cm^−1^ for ether stretching (C–O) (Figure 2) [31].

The spectrum obtained for porcine gelatin (Figure 2A,B) shows vibrational bands in the region of 1636 cm^−1^ for the asymmetric and symmetrical elongation of the amide I bond (C=O), bands at 1617 cm^−1^ corresponding to amide II (NH), and at 3549 cm^−1^ and 3412 cm^−1^ concerning stretches of (NH) and (OH) bonds, respectively, which can form hydrogen bonds with the carbonyl group of the peptide bond [22,32]. Sodium alginate (Figure 2C,D) showed vibrations in the region of 1589–1466 cm^−1^, which corresponded to the presence of the carbonyl group (C=O).

Characteristic bands in the formulation LAEG (Figure 2A) were observed at 3008 cm^−1^, referring to dimers of secondary amides (NH) in s-cis or s-trans conformations [32], at 1235 cm^−1^ for phosphate-bearing compounds, and at 1162–916 cm^−1^ attributed to microbial cell wall carbohydrates [33]. Furthermore, the bands at 2926 cm^−1^ and 1744 cm^−1^ may be associated with the CH_2_ elongation of fatty acids from the probiotic’s lipid membranes and the presence of corn oil [34], used as the oil phase in O/W emulsification encapsulation.

It can be seen that there was a chemical interaction between the gelatin and Tween 20 surfactant after attenuating the absorption bands in the gelatin for the amide group (3552 cm^−1^ and 3416 cm^−1^) and vibrational stretching at 2926 cm^−1^ and 2853 cm^−1^ for Tween 20.

Characteristic bands of the chemical constituents present in the microorganism and the oil for the encapsulated LPEG (Figure 2B) were observed at 3008 cm^−1^, 2926 cm^−1^, 1744 cm^−1^, 1239 cm^−1^, and 1166–1030 cm^−1^, showing the presence of the NH, CH_2_, P=O, CO, CH, and C–C(=O)–C groups [32,33]. Furthermore, the presence of gelatin-related bands at 3549 cm^−1^, 3479 cm^−1^, and 3412 cm^−1^ (N–H) and vibrational stretching of the bands at 2926 cm^−1^ and 2853 cm^−1^ for Tween 20. These results confirm the chemical interactions in the system, indicating that the probiotics were encapsulated in porcine gelatin.

On the other hand, it was impossible to observe characteristic bands of the chemical constituents present in the probiotic in the LAEA formulation spectra (Figure 2C) such as the components of the microbial structure. It was only possible to visualize the attenuation of the Tween 20 (2923 cm^−1^ and 2856 cm^−1^) and alginate bands (3545–3239 cm^−1^ and 1469 cm^−1^), but without stretching or the formation of new bands. Similarly, *L. plantarum* in alginate (Figure 2D) also did not present characteristic bands of the chemical groups present in the probiotic, only displacements of alginate characteristic bands (3384 cm^−1^, 3273 cm^−1^, 3173 cm^−1^, and 3091 cm^−1^), however, not attenuated. It is assumed that the low chemical interaction between the system’s constituents for LAEA and LPEA did not favor incorporating microorganisms, which were more exposed during the encapsulation process, resulting in a loss of viability.

#### 3.1.5. X-ray Diffraction

The diffractograms showed structures of a semi-crystalline nature, with noise characterizing amorphous regions and defined peaks in the crystalline regions for porcine gelatin (PG) 2θ = 22.07°, and alginate (AG) 2θ = 19.94°, 20.79°, 23.30°, 25.51°, 28.46°, 31.40°, 35.51°, 36.34° (Figure S2—Supplementary Materials). De Oliveira et al. [22] also observed these characteristic peaks.

Those encapsulated in gelatin (LAEG and LPEG) presented a single translocated peak at 2θ = 19.86° and 20.65° (Figure S2—Supplementary Materials), which may be related to the presence of the chemical interaction of porcine gelatin with the probiotic nucleus, evidenced in the result obtained by FTIR analysis. As observed for the crude polymer, the alginate microparticles (LAEA and LPEA) obtained characteristic crystalline peaks. These data reaffirm the results found previously for the alginate encapsulates. The shrinkage and irregularity of the structure were noticeable as well as low chemical interaction between the components used.

### 3.2. Enumeration of Viable Cells and Encapsulation Efficiency

After encapsulation using gelatin, *L. acidophilus*-LAEG showed a reduction of 1.2 Log CFU/g in viability, corresponding to an EE of 89.6 ± 4.2% (Table 1). A similar EE value was found for *L. plantarum*-LPEG (81.1%), with a reduction of 2.4 Log CFU/g. Using an Ultra-Turrax dispenser for homogenization during the encapsulation process represents a risk factor since the homogenization can cause physical damage to the microbial cells. However, the high EE values indicated that O/W emulsification is compatible with probiotic encapsulation. Similarly, Matos-Jr et al. [14] did not verify a significant reduction in counting viable *L. rhamnosus* 64 and *L. paracasei* BGP1 cells when submitted to homogenization using an Ultra-Turrax at 5500 and 7000 rpm.

The EE results using gelatin corroborate data obtained from SEM, in which it was possible to observe the probiotic cells arranged under gelatin-based particle structures (LAEG and LPEG). Furthermore, the FTIR results showed the chemical interactions between the raw agents (gelatin and Tween 20) and the microorganisms, indicating the protection of probiotics.

According to Sagiri et al. [35], studies with excellent encapsulation efficiency present percentages above 80%. In addition, the microencapsulation procedure using gelatin resulted in high efficiency compared to that found by other authors. When evaluating the survival of the *Bifidobacterium adolescentis* 15703T probiotic encapsulated in type A porcine gelatin (13%) coated with 1% alginate by the calcium gelation technique, Annan et al. [36] obtained an EE of 41 to 43% for the internal phase method and 30% for the external phase. Khalil et al. [37] reported that the extrusion method’s encapsulation of *B. pseudocatenulatum* G4 in Type B gelatin resulted in an EE of only 45.9%.

However, a drastic decrease in the viability of *L. acidophilus* and *L. plantarum* was observed when using alginate as an encapsulating agent, with an encapsulation efficiency < 1.0% (Table 1). Alginate is commonly used as an encapsulating material for microbial cells, forming more porous matrices susceptible to disintegration in excess monovalent ions and calcium-chelating agents [3]. Ester et al. [38] showed that encapsulation with alginate did not guarantee the survival of *L. salivarius* spp. *salivarius*, with a 60% loss of viability due to the porous surface.

Therefore, it is suggested that sodium alginate did not protect the probiotic bacteria in this study. Thus, the viability of microorganisms was compromised by exposure to factors such as oxygen, temperature, and shear tension during homogenization.

### 3.3. Dispersibility

The dispersibility test showed that the LAEG and LPEG obtained a water solubilization of 69.9 ± 9.7% and 69.0 ± 8.4% (*p* > 0.05). Solubility directly influences food application, favoring (or not) acceptance. The laser diffraction results showed that LAEG and LPEG had smaller particle diameters associated with the physical and chemical characteristics of the encapsulating agent, possibly influencing the good solubility of the particles.

It is important to highlight that gelatin is a biodegradable protein material derived from the partial hydrolysis of collagen. It is an excellent choice as a wall material due to its high water solubility, emulsifying, and thickening ability [39].

### 3.4. Thermal Analysis

**Figure 3:**
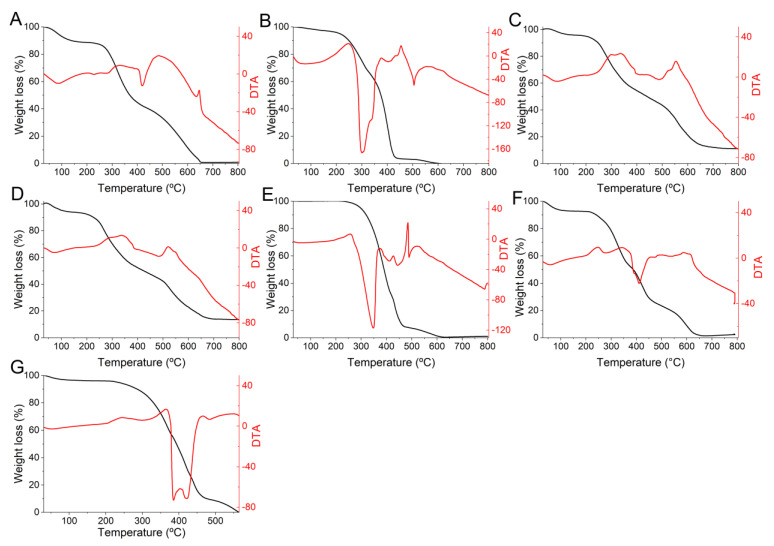
Thermograms and DTA of the encapsulating and encapsulated materials obtained by O/W emulsification. (**A**) Porcine gelatin; (**B**) Tween 20; (**C**) *L. acidophilus*; (**D**) *L. plantarum*; (**E**) corn oil; (**F**) LAEG—*L. acidophilus* in gelatin; (**G**): LPEG—*L. plantarum* in gelatin.

The porcine gelatin used as an encapsulating matrix lost considerable mass (58%) at 420 °C in melting events (Figure 3A), which is associated with the degradation and modification of gelatin chains. In comparison, 68% decomposition occurred at 505 °C of DTA due to the breakdown of the more thermally stable structures [40].

These results differ from those found by Khodaei et al. [41], who observed endothermic peaks at 66–104 °C and at 232–268 °C when evaluating the ability of *L. plantarum*, *L. casei*, and *S. boulardii* to survive in edible films based on gelatin and low methoxyl pectin (LMP). However, the gelatin used in this study was Type B (bovine gelatin).

Other constituent materials also partially lost mass in the melting event such as Tween 20 surfactant (56%) (Figure 3B) and corn oil (68%) (Figure 3E), with a final decomposition with TG of 94–86% at temperatures from 420 °C to 445 °C (DTA). However, microorganisms were affected by the melting event at temperatures ranging up to 478 °C. *L. acidophilus* showed a loss of 55% and *L. plantarum* of 56% (TG). This can be explained by the degradation of cellular constituents of probiotics such as proteins, lipids, and polysaccharides [13]. Their final decompositions occurred at temperatures corresponding to a mass loss (TG) of 68% and 61% at 555 °C and 522 °C, respectively.

The mass loss was considerably lower (4%) for gelatin encapsulates up to 65 °C (Figure 3F,G). As the events started at ambient temperatures, this loss was assumed to be due to water elimination from the samples. The mass loss of the encapsulates then occurred gradually after these initial endothermic events. Melting events conditioned the encapsulated samples’ highest mass losses, ranging from 58 to 69% for LAEG (Figure 3F) and LPEG (Figure 3G), respectively. This may be related to the components’ degradation in the wall material. The exothermic decomposition peak for LPEG was 559 °C and 616 °C for LAEG.

### 3.5. Evaluation of the Stability of Encapsulated Probiotics during Storage

**Figure 4:**
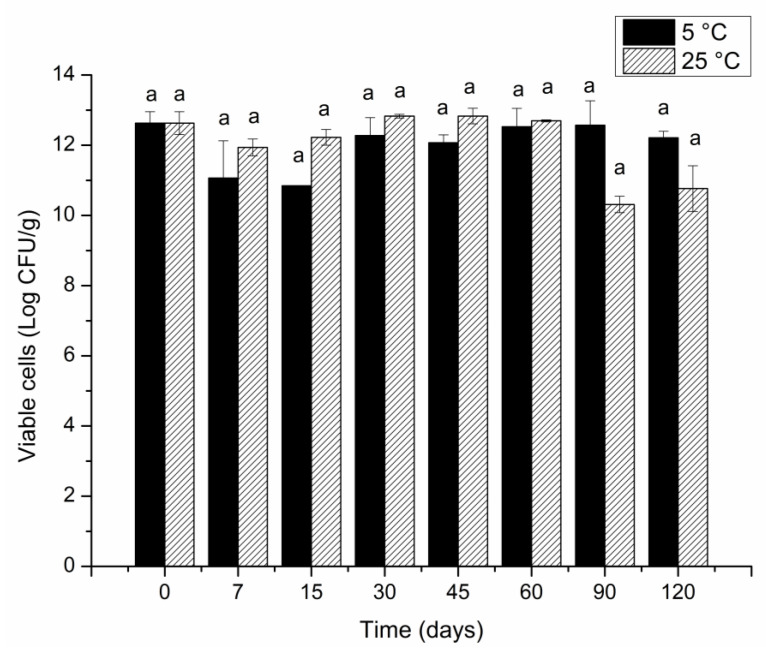
Viability of microparticles containing *L. acidophilus* in porcine gelatin by O/W emulsification (LAEG) during 120 days of storage at 25 °C and 5 °C. ^a^ Equal letters in the same temperature storage indicate no statistically significant difference (*p* > 0.05) at different times.

According to the results (Figure 4), encapsulation by O/W emulsification using gelatin efficiently protected the probiotic in the two conditions studied. It is possible to observe good viability throughout the evaluated period. At the end of the studied period, the LAEG encapsulate showed the viability of 12.2 ± 0.1 Log CFU/g at 5 °C and 10.7 ± 0.6 Log CFU/g at 25 °C.

The data showed that the encapsulates maintained superior viability even at room temperature (25 °C) compared to other studies [4,41] that used gelatin as an encapsulating agent. Paula et al. [4] found viability of 7.6 Log CFU/g after 45 days of storage at 8 °C and −18 °C by microencapsulating *L. plantarum* by double emulsification followed by complex coacervation using gelatin and gum Arabic.

**Figure 5:**
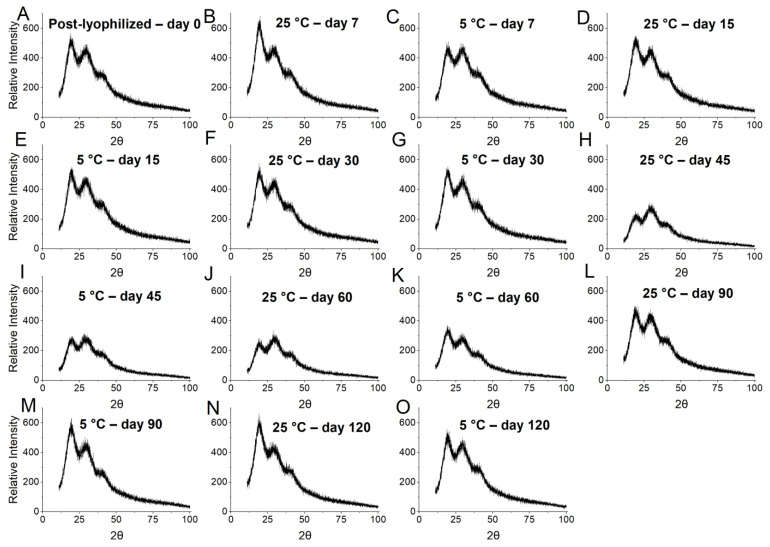
Diffractograms of the gelatin microparticles (LAEG) during storage at different temperatures. (**A**) Post-lyophilized–day 0; (**B**) 25 °C–day 7; (**C**) 5 °C–day 7; (**D**) 25 °C–day 15; (**E**) 5 °C–day 15; (**F**) 25 °C–day 30; (**G**) 5 °C–day 30; (**H**) 25 °C–day 45; (**I**) 5 °C–day 45; (**J**) 25 °C–day 60; (**K**) 5 °C–day 60; (**L**) 25 °C–day 90; (**M**) 5 °C–day 90; (**N**) 25 °C–day 120; (**O**) 5 °C–day 120.

The X-ray diffraction technique was used to evaluate the effects of storage on the porcine gelatin-based encapsulate structures. The diffractograms showed (Figure 5) that the formulations maintained their semi-crystalline nature for the two temperatures assessed throughout the storage period. The presence of noise that characterizes amorphous areas is notable, and the presence of defined peaks indicates crystalline areas at 19° and 20°.

It is possible to notice changes in the structure of the microparticles at 45 (Figure 5H,I) and 60 days (Figure 5J,K) of storage. Amorphous and crystalline areas were attenuated under these conditions. However, it can be noted that translocation of the crystalline peak to 2θ = 28.5° only occurred on day 60 at room temperature (Figure 5J). This structural slowing down may likely suggest the onset of disturbances in intramolecular associations, possibly due to the temperature and a long storage period [42].

More pronounced changes in the structure were observed for both storage conditions from day 90 (Figure 5L,N), with the presence of crystalline peaks at 2θ = 19°, 20°, 26°, 27°, 28°, 29°, 37°, and 39°. Crystalline peaks were observed at 2θ = 18°, 19°, 20°, 29°, 30°, 31°, 34°, and 40° at 120 days (Figure 5M,O), possibly indicating agglomeration and recrystallization of the structure. In addition, it is possible to observe an increase in the intensity of the amorphous regions of the structure. Amorphous regions of the matrices are generally more soluble and hygroscopic, thus facilitating agglomeration of the structure.

Agglomeration and crystallization can lead to a release of components encapsulated in the matrix [43], which in this case could culminate in the release of probiotics trapped in the porcine gelatin structures. However, it is important to highlight that these were not enough to compromise the probiotic viability over the 120 days of storage, despite the structural changes mentioned.

An essential aspect of maintaining probiotic viability is carefully selecting the encapsulation technique and materials [3]. The good stability obtained in the present study can be attributed to the formation of microcapsules by O/W emulsification using porcine gelatin. This protection can be explained by forming the gelatin network associated with the hermetic filling of the oil droplets [18]. Thus, the microparticles obtained in this study are stable and have potential applications in the food industry.

Finally, lyophilization is a drying strategy used to manufacture food products, which enables the preservation of probiotic cells [44]. This technique can diversify the application in food matrices without restricting emulsified foods, allowing for commercialization in powder, capsules, and chewable tablets, among others [2].

## 4. Conclusions

Microencapsulation of *L. acidophilus* and *L. plantarum* by O/W emulsification using gelatin has been proven to be a suitable technology, resulting in higher encapsulation efficiencies. In addition, the present work showed that gelatin-based microparticles presented favorable characteristics for protecting and maintaining probiotic viability during 120 days of storage at 5 °C and 25 °C. Therefore, the results obtained in the present study are important for the food industry as support for using O/W emulsification to apply probiotics in food matrices.

## Figures and Tables

**Table 1:** Mean diameter, polydispersity index, viable cell count before and after encapsulation, and the probiotic encapsulation efficiency (EE%).

Formulation	Mean Diameter (µm) *	Polydispersion Index *	Viable Cell Count (Log CFU/g)		EE% *
			Before	After	
LAEG	26.08 ± 1.74 ^a^	0.5 ± 0 ^a^	12.1 ± 1.0	10.9 ± 0.9	89.6 ± 4.2 ^a^
LPEG	21.56 ± 4.17 ^a^	0.6 ± 0.1 ^a^	12.3 ± 1.4	9.9 ± 0.8	81.1 ± 9.7 ^a^
LAEA	5.24 ± 1.32 ^b^	0.1 ± 0.1 ^b^	12.8 ± 0.0	<1	<1 ^b^
LPEA	5.52 ± 0.45 ^b^	0.1 ± 0 ^b^	12.7 ± 0.2	<1	<1 ^b^

LAEG—*L. acidophilus* in gelatin; LPEG—*L. plantarum* in gelatin; LAEA—*L. acidophilus* in alginate; LPEA-LPEA—*L. plantarum* in alginate. * Values expressed as mean ± standard deviation (*n* = 3). Equal lowercase letters in the same column indicate no statistically significant difference (*p* > 0.05), based on ANOVA and Tukey’s post-test.

## Data Availability

The data presented in this study are available on request from the corresponding author.

## Supplementary Materials

The following supporting information can be downloaded at: https://www.mdpi.com/article/10.3390/foods12020252/s1, Figure S1: Zeta potential of encapsulates obtained by O/W emulsification; Figure S2: X-ray diffractograms of the powder particles of encapsulates obtained by O/W emulsification.
