# Supplementary material for: Microencapsulation of Probiotics by Oil-in-Water Emulsification Technique Improves Cell Viability under Different Storage Conditions
Source: Foods. 2023 Jan 5;12(2):252. doi: 10.3390/foods12020252 (PMC9857835; doi:10.3390/foods12020252)
Supplement: Supplementary file 1 [file foods-12-00252-s001.zip › Supplementary material S.1.pdf]

Supplementary material S.1

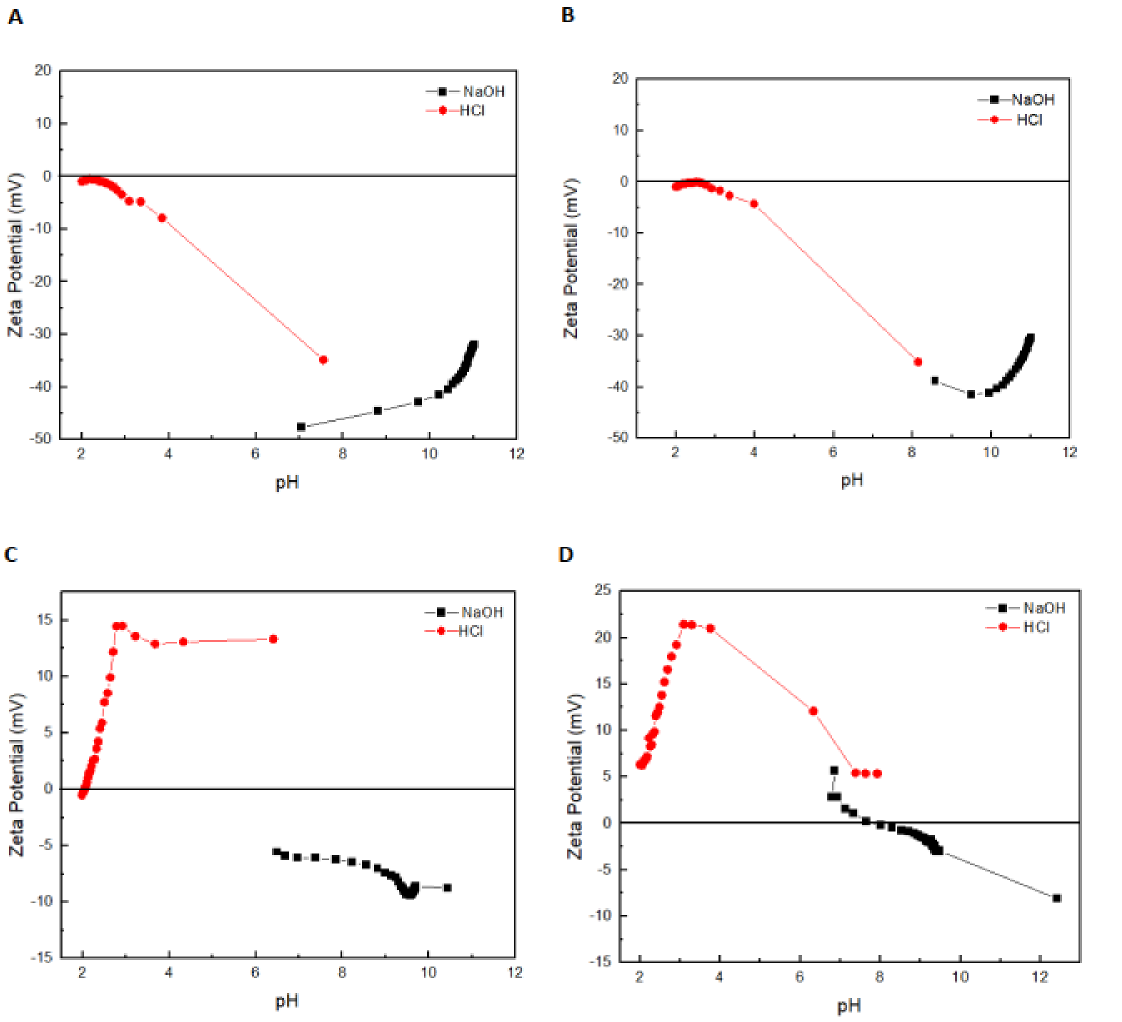

Figure S1. Zeta Potential of encapsulates obtained by O/W emulsification. (**A**) LAEA: *L. acidophilus* in alginate; (**B**) LPEA: *L. plantarum* in alginate; (**C**) LAEG: *L. acidophilus* in gelatin; (**D**) LPEG - *L. plantarum* in gelatin.
