# Supplementary material for: Microencapsulation of Probiotics by Oil-in-Water Emulsification Technique Improves Cell Viability under Different Storage Conditions
Source: Foods. 2023 Jan 5;12(2):252. doi: 10.3390/foods12020252 (PMC9857835; doi:10.3390/foods12020252)
Supplement: Supplementary file 1 [file foods-12-00252-s001.zip › Supplementary material S.2.pdf]

Supplementary material S.2

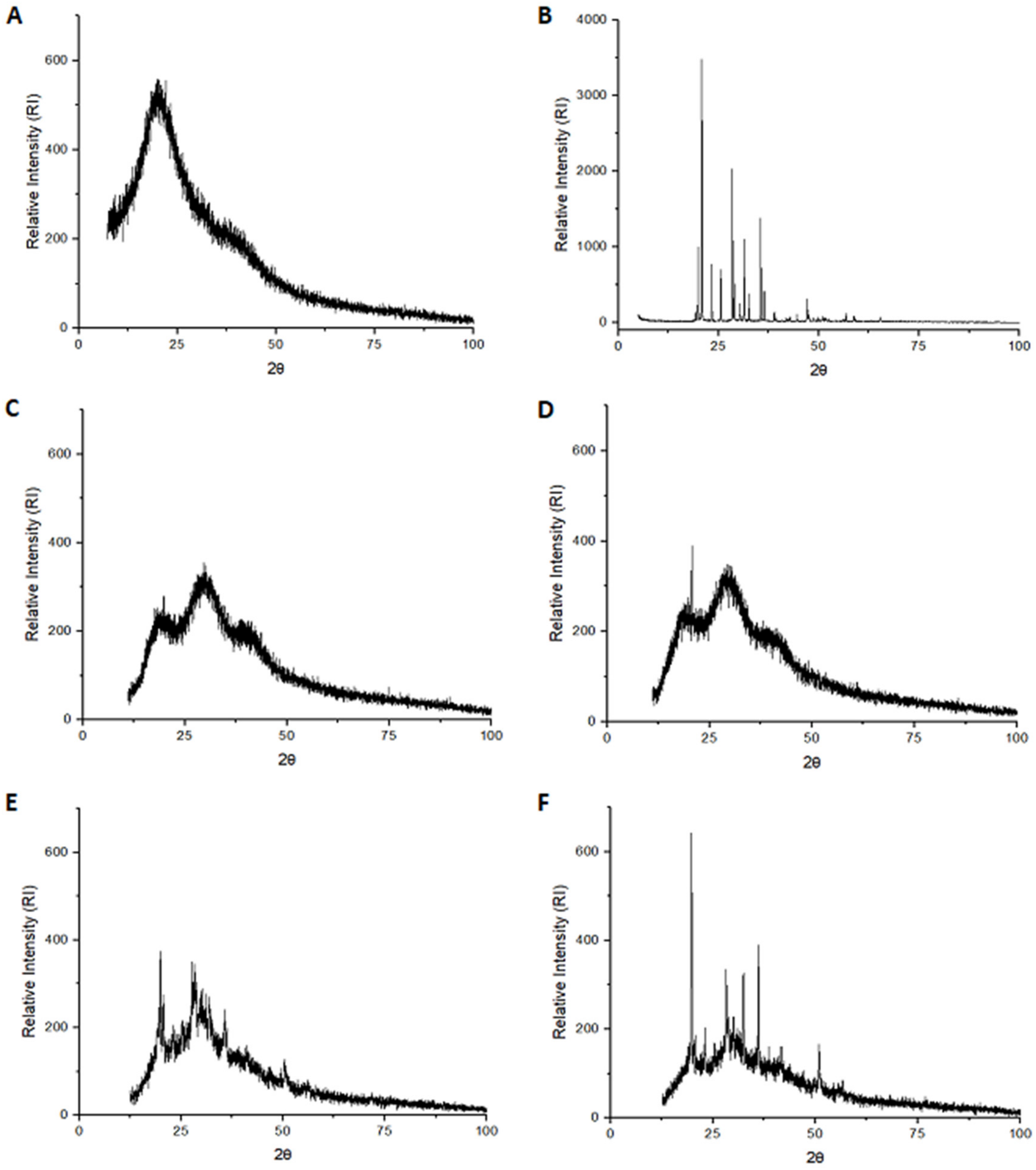

Figure S2. X-ray diffractograms of the powder particles of the encapsulates obtained by O/W emulsification. (**A**) porcine gelatin; (**B**) sodium alginate; (**C**) LAEG - *L. acidophilus* in gelatin; (D) LPEG - *L. plantarum* in gelatin; (**E**) LAEA - *L. acidophilus* in alginate; (**F**) LPEA - *L. plantarum* in alginate.
